# A Bayesian framework for happiness, health, and psychological well-being

**DOI:** 10.3389/fpsyg.2026.1778763

**Published:** 2026-07-09

**Authors:** Federica Mauro

**Affiliations:** Independent Researcher, Rome, Italy

**Keywords:** active inference, health, homeostasis, mindfulness, normativity (Canguilhem), psychological richness, psychotherapy, well-being and happiness

## Abstract

Classical accounts of well-being have largely been grounded in homeostasis, conceptualizing well-being as the regulation of affective states around defended set points. More recent Bayesian and active inference approaches similarly characterize living systems as predictive regulators that maintain viable states by minimizing expected surprise. Building on these perspectives, this article proposes an integrative Bayesian framework in which well-being is understood not as a fixed scalar quantity, but as a dynamically maintained viability corridor defined by hierarchically organized prior expectations about preferred affective and interoceptive states. Classical accounts of well-being have largely been grounded in homeostasis, conceptualizing well-being as the regulation of affective states around defended set points. More recent Bayesian and active inference approaches similarly characterize living systems as predictive regulators that maintain viable states by minimizing expected surprise. Building on these perspectives, this article proposes an integrative Bayesian framework in which well-being is understood not as a fixed scalar quantity, but as a dynamically maintained viability corridor defined by hierarchically organized prior expectations about preferred affective and interoceptive states. Within this framework, homeostasis and allostasis are viewed as complementary aspects of the same regulatory architecture. Homeostasis refers to the maintenance of vital variables within viable bounds, whereas allostasis denotes the predictive processes through which such stability is achieved under changing conditions. Well-being is interpreted as the phenomenological expression of these regulatory dynamics, closely related to core affect while remaining analytically distinct from the mechanisms that sustain it. At the event level, affective experience is described in information-theoretic terms. Arousal is formalized as information gain arising from the interaction between prediction error and prior uncertainty, whereas valence follows an inverted-U relationship consistent with the Wundt curve. Drawing on the Central Limit Theorem, the framework further suggests that moderate and metabolically efficient regimes are both statistically prevalent and hedonically preferred, helping to explain why organisms tend to gravitate toward intermediate levels of stimulation. Health is reconceptualized as metastable attunement: a dynamic balance between stability and exploration sustained through flexible precision allocation. Within this perspective, valence functions as a control signal that guides precision tuning across timescales, enabling organisms to maintain viable states while adapting to changing environmental demands. Different dimensions of the good life can then be understood as expressions of this broader regulatory architecture. Happiness reflects the experience that regulation is proceeding better than expected at an acceptable energetic cost; eudaimonia emerges from the alignment of action with identity-level priors, values, and long-term goals; and psychological richness reflects epistemic exploration that expands the agent’s generative model. Accordingly, mindfulness and psychotherapy are interpreted as complementary routes to adaptive regulation, promoting greater flexibility and integration across levels of the self-model. Situated within health neuroscience, the proposed framework provides a theoretically grounded basis for future research at the intersection of computational neuroscience, clinical psychology, and well-being science.

## Introduction

Subjective well-being (SWB)— a concept that often overlaps with that of “happiness”—captures how people judge and feel about their lives, drawing on both what happens to them and the psychological resources they bring to those circumstances (Diener, 2006; Durand, 2015). External conditions such as income and relationships, together with internal factors like goals, outlook, and coping capacities, shape these appraisals; negative evaluations typically prompt attempts to change one’s situation, while positive evaluations support maintenance or further investment (Diener, 2006; Durand, 2015).

Clarifying SWB benefits from distinguishing it from, yet relating it to, adjacent constructs. For example, health is notoriously hard to pin down beyond the WHO’s broad definition, contemporary accounts contrast a biomedical view—health as absence of abnormality—with a functional view that emphasizes “the strength to be” and to meet challenges, alongside role fulfillment, autonomy, optimism, and, for some, spirituality (for a critical perspective see Mauro et al., 2025b). Crucially, both personal adaptation and socio-economic or environmental policy shape health trajectories (Misselbrook, 2014; Christoforou et al., 2024). Quality of life (QoL), as defined by the WHO, refers to people’s culturally situated perceptions of their position in life given their goals and expectations, influenced by physical health, psychological state, independence, social ties, and environment (WHOQOL Group, 1995). SWB overlaps with QoL but is usually specified as comprising three strands: (i) global life evaluations, (ii) current affective states, and (iii) eudaimonia—meaning and purpose (Diener, 2006; Durand, 2015; Marks, 2024), and this tripartite framing, aligning cognitive judgments, affect balance, and purpose, sits comfortably within contemporary, multidimensional well-being frameworks (Bautista et al., 2023).

Other accounts extend trait-based perspectives by adopting a systems view in which psychological, social, and biological processes jointly contribute to maintaining SWB within a functional range. Within homeostatic models, these regulatory processes act, under ordinary conditions, to restore SWB following perturbations, maintaining it within a relatively narrow, individually characteristic range (Cummins, 2016). When stressors are intense, prolonged, or repeated, however, this homeostatic regulation may become less efficient, resulting in slower or only partial recovery. These accounts build on the biological concept of homeostasis — the organism’s capacity to preserve internal stability through self-regulating negative feedback mechanisms (Cannon, 1932) — and propose that individuals fluctuate around an idiosyncratic “set point,” buffered by psychological and social resources.

By contrast, allostatic approaches emphasize adaptive regulation through change rather than constancy. Allostasis refers to the process by which regulatory systems adjust their parameters — including set points — in response to environmental demands (Sterling and Eyer, 1988). Within this framework, repeated or chronic stressors can lead not only to temporary deviations from equilibrium but to more enduring shifts in regulatory dynamics. The cumulative physiological and psychological burden of these adjustments is captured by the concept of allostatic load, which has been linked to increased morbidity and mortality risk (McEwen and Stellar, 1993).

In this context, Dowling and Yap (2006) proposed a behavioral model in which human choices aim to maintain or restore a multidimensional equilibrium. The preferred set point integrates physical, emotional, psychological, economic, and environmental dimensions and is experienced as safety, trust, close relationships, and predictable, supportive work and social settings. Departures from this equilibrium reduce well-being and prompt corrective action. While the model is primarily homeostatic in positing a tendency toward equilibrium, it also incorporates allostatic elements, insofar as severe or prolonged perturbations may lead to a redefinition of the baseline or to lasting damage. In line with this, higher allostatic load corresponds to greater morbidity and mortality risk. Positive shocks (e.g., marriage, promotion) may temporarily elevate well-being as the system stabilizes at a higher level, whereas negative shocks (e.g., job loss, divorce, illness) depress well-being and trigger coordinated physical, emotional, behavioral, and cognitive efforts to regain balance.

More generally, these accounts formalize well-being as a dynamic regulatory process. From a homeostatic perspective, well-being reflects the system’s tendency to return to a characteristic set-point range following perturbation. From an allostatic perspective, however, well-being also depends on the system’s capacity to adapt its regulatory parameters to changing conditions, often at a cost. In this sense, well-being can be understood as the felt signature of proximity to an organism–environment equilibrium: perturbations displace the system from this range, while regulatory processes act either to restore it (homeostasis) or to recalibrate it (allostasis), with associated costs that forecast downstream health and mortality risk. Importantly, following Ramsay and Woods (2014), homeostasis and allostasis should not be viewed as competing models but as complementary aspects of physiological regulation. Homeostasis refers to the maintenance of vital physiological variables within viable ranges, whereas allostasis refers to the adaptive adjustments of regulatory systems that enable organisms to achieve this stability under changing environmental conditions.

Against this theoretical backdrop, at the center of Subjective Well-Being Homeostasis Theory lies the construct of Homeostatically Protected Mood (HPMood; Cummins, 2010; Davern et al., 2007). HPMood builds on Russell’s (2003) notion of core affect, defined as a “neurophysiological state consciously accessible as the simplest raw (non-reflective) feeling evident in moods and emotion” (p. 148). Core affect is thus described as consciously experienced yet non-cognitive and non-reflective, continuously present as a background condition, objectless and causeless, and situated at the core of all emotion-laden events. In a subsequent revision, Russell (2009) refined this view by distinguishing a prototypical emotional episode, which is “directed at something,” from core affect, which is “not necessarily directed at anything” (p. 7), while allowing that core affect can become object-directed.

Within Cummins’ theory of SWB, HPMood both constitutes the affective component of SWB and functions as the steady-state affective set point that homeostatic processes act to maintain. Empirical support was first provided by Davern et al. (2007), who operationalized HPMood via the affects happy, contented, and excited, and showed that these indicators explained 64% of the variance in SWB, suggesting a dominant affective contribution. On this basis, Cummins (2010) argued—on evolutionary grounds—that survival has been favored among individuals whose HPMood stabilizes within an optimal range of approximately 70–80 points pleasant/positive, representing the most adaptive level of affective tone.

A more recent contribution comes from Marks (2024), who advanced a Homeostasis Theory of Well-Being that treats well-being as the emergent property of a modular homeostatic system. He proposes 16 interacting modules — spanning affect, physical health, social connectedness, and other life domains — which together act to stabilize overall well-being. These modules are supported by psychological resources such as self-esteem, perceived control, and optimism, which function as buffers against external stressors. On statistical grounds, Marks further argues that, by the Central Limit Theorem, any multi-input homeostatic system will tend to produce outputs that approximate a Gaussian distribution, implying that most individuals’ well-being will cluster around a characteristic range rather than drift arbitrarily.

Despite these contributions, there is still no shared framework that reconciles classical homeostatic and allostatic models of subjective well-being, also with more recent accounts of well-being that emphasize its placement within a predictive regulation framework (see for example Miller et al., 2022). In this context, the aim of the present article is to propose an integrated perspective on well-being, in which subjective well-being is understood not only as a defended set point, but as the emergent product of ongoing regulatory processes that include both homeostatic stabilization and adaptive, predictive adjustment, allowing to reinterpret well-being, health and happiness within the same unified framework.

## A Bayesian theoretical account of well-being as homeostatic core affect

In contemporary theoretical neuroscience, homeostasis is no longer treated simply as the passive maintenance of internal variables within fixed bounds. It is reframed as a problem of self-organization under uncertainty. On this view, to stay alive is to preserve a set of statistically reliable relations with the environment — to keep occupying a characteristic region of state space despite constant perturbation. This requires maintaining a boundary between “what is me” and “what is out there,” and this boundary is formalized as a Markov blanket (Kirchhoff et al., 2018; Hipólito et al., 2021).

**FIGURE 1:**
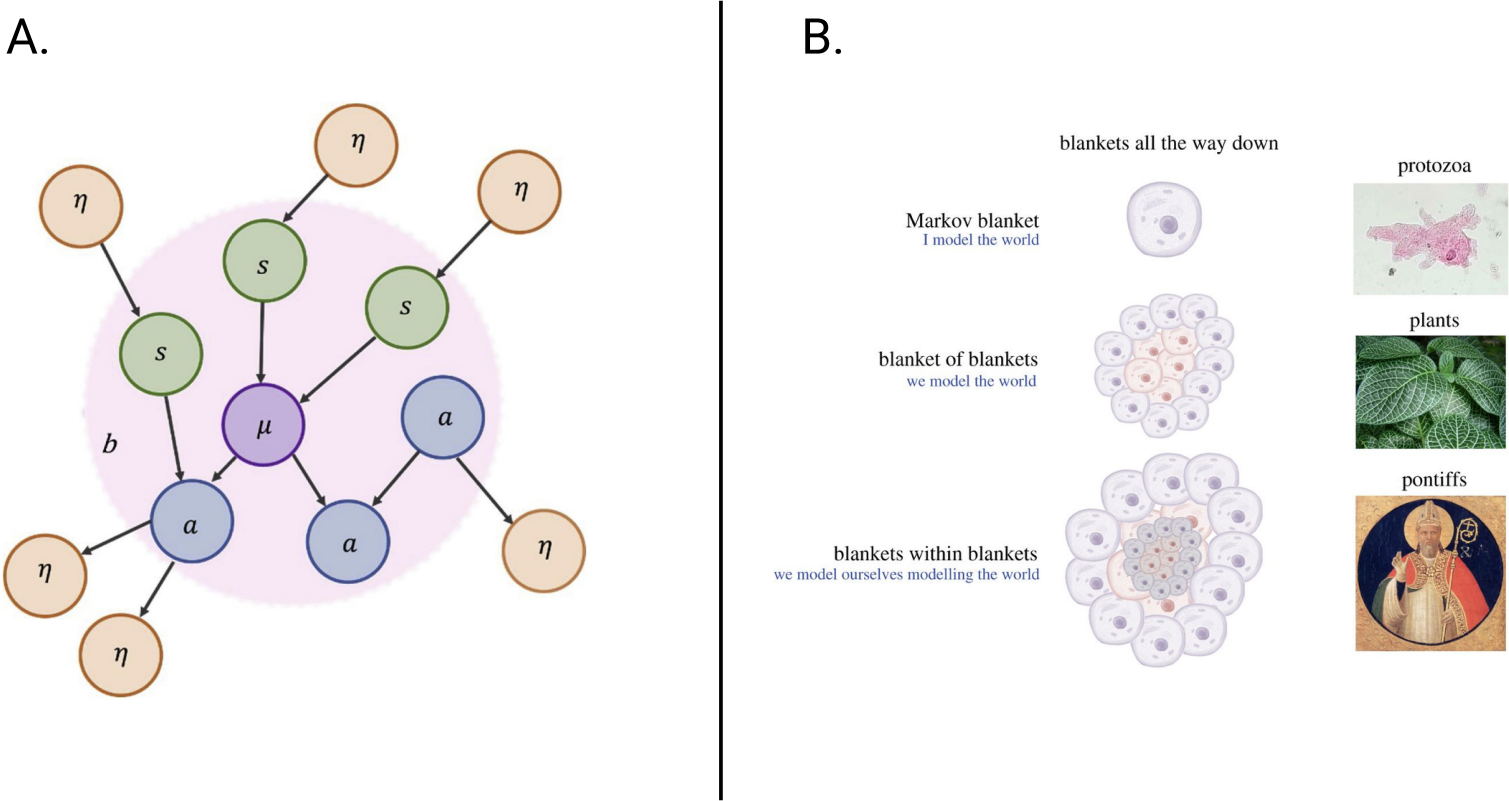
**(A)** Markov blanket. Markov blankets characterize open systems that exchange matter, energy, or information with their surroundings. The Figure shows the Markovian partition of a system (or set of states) into internal states (μ), blanket states—subdivided into active states (a) and sensory states (s)—and external states (η). Internal and external states are conditionally independent given the blanket states. In the associated directed graphical model, variables are conditionally independent when there is no connecting route between them and they share parents; arrows indicate directed dependencies from “parents” to “children.” Adapted from Hipólito et al. (2021). **(B)** Schematic depiction of nested Markov blankets. The recursive organization of Markov blankets allows autonomous systems to emerge across multiple scales, from cells to organisms and social systems. In this view, higher-order forms of organization arise through the integration of lower-level autonomous units, yielding a hierarchy of self-organizing and self-maintaining systems. Adapted from Kirchhoff et al. (2018).

A Markov blanket partitions the world into internal states and external states, with two mediating sets: sensory states, through which the external world influences the internal, and active states, through which the internal system acts back on the world. Conditional independencies across the blanket mean that internal states can be interpreted as inferring (i.e., forming probabilistic beliefs about) the hidden causes of sensory input, while active states can be interpreted as interventions that change those causes (see Figure 1A). Crucially, living systems are hierarchically nested: cells inside organs, organs inside organisms, organisms inside relational and ecological niches. Because of this nesting, Markov blankets are multiscale and need not coincide with a single biological membrane. Autonomy, under this view, is not an all-or-nothing property of a sharply bounded body, but an emergent, scale-relative achievement of self-sustaining blankets embedded within blankets (see Figure 1B; Kirchhoff et al., 2018; Hipólito et al., 2021).

The Free Energy Principle provides the optimization logic underlying this framework. It proposes that any system that maintains its integrity over time — that is, any system that does not dissolve into its environment — must, on average, minimize an upper bound on the surprisal (i.e., improbability) of its sensory states. This bound is known as variational free energy (Friston, 2010; Buckley et al., 2017; Parr and Friston, 2019). In practice, minimizing free energy involves reducing the mismatch between predicted and observed sensory inputs, thereby minimizing prediction errors and preserving the organism’s capacity to maintain adaptive engagement with its environment.

Within this framework, perception and action are two complementary aspects of the same process. On the one hand, internal states are updated to better predict incoming sensory signals (perceptual inference). On the other hand, actions are selected so as to sample sensory inputs that match those predictions (active inference). Regulation is therefore not merely reactive but inherently anticipatory: the system does not simply correct deviations, but continuously predicts and counteracts them. In this sense, classical homeostasis can be reinterpreted as a form of predictive regulation, closely aligned with the concept of allostasis.

From this perspective, the brain can be understood as a Bayesian system. Internal states encode probabilistic beliefs about hidden causes of sensory input; perception approximates Bayesian inference; and action implements active inference by selecting policies expected to minimize future free energy (Friston, 2010; Parr and Friston, 2019). Predictive processing provides a mechanistic account of this architecture in hierarchical neural systems: higher levels generate predictions about lower levels, lower levels return prediction errors, and the system iteratively updates beliefs or acts on the environment to reduce these errors.

The concept of the Markov blanket defines the boundary that separates the system from its environment while enabling structured exchange between the two. Within this boundary, inference and control become possible for an embodied agent. The Free Energy Principle, in turn, specifies the normative imperative guiding this process: self-maintenance. To maintain its Markov blanket is, in effect, for the system to “self-evidence” — that is, to act in ways that keep generating the sensory states consistent with its continued existence, thereby accumulating evidence for the hypothesis that it is the kind of system it is (Hohwy, 2013; Hipólito et al., 2021; Parr and Friston, 2019).

Under this view, homeostasis is not replaced but reframed as inherently inferential and predictive: allostasis corresponds to predictive control—implemented through belief updating and action selection—while homeostasis corresponds to the emergent stability achieved through this process (Friston, 2010; Pezzulo et al., 2015; Krupnik, 2024). As noted in the Introduction (following Ramsay and Woods, 2014), these are not competing models but complementary aspects of the same regulatory architecture.

### Toward a multilevel predictive model of well-being

Within this predictive framework—where allostatic regulation supports homeostatic stability through anticipatory control (Ramsay and Woods, 2014; Krupnik, 2024)—the brain is not a passive system reacting to stimuli. Rather, it continuously generates top-down predictions about perception, bodily states, and action. Incoming sensory signals — including interoceptive signals from the body — are evaluated against these predictions. Any mismatch, or prediction error, is then used to update the internal model, improving the accuracy and efficiency of future predictions. Perception, in this sense, is not a direct readout of the external world but the brain’s best current estimate, continuously revised by error signals. Likewise, action is not a downstream response to perception, but is already embedded within the predictive process as part of how the organism prepares to regulate itself in context.

According to Barrett (2017), the brain continuously anticipates the body’s needs — metabolic, autonomic, endocrine, and immune — and adjusts internal states in advance to meet them efficiently. Brain regions traditionally labeled as “emotion centers,” including the anterior insula, anterior cingulate cortex, ventromedial prefrontal cortex, amygdala, hypothalamus, and periaqueductal gray, are better understood as visceromotor control hubs. Rather than triggering discrete emotions, these regions issue predictive regulatory commands, coordinate physiological and behavioral responses, and manage the body’s energy resources. Emotional experience, in this framework, is inseparable from this ongoing predictive regulation: affect — defined along the dimensions of valence (pleasant–unpleasant) and arousal (activated–deactivated) — reflects the conscious experience of these regulatory processes.

From an interoceptive perspective, bodily regulation can then be formalized as a Bayesian control loop. Within this loop, the central nervous system must continuously (i) infer the current internal state of the body from noisy interoceptive signals, (ii) select regulatory actions to maintain physiological stability, and (iii) anticipate how internal states will evolve over time as a result of both intrinsic dynamics and those actions. Crucially, the goal is not merely to correct deviations after they occur, as in classical homeostasis, but to anticipate and prepare for future needs — consistent with predictive regulation and allostasis (see Khalsa et al., 2018; Petzschner et al., 2021).

Accordingly, interoception can be understood as a form of Bayesian inference in which the brain estimates hidden bodily states (e.g., blood pressure or osmolarity) by combining incoming sensory signals with prior expectations about viable physiological conditions. This process yields a posterior belief about the current state of the body. The relative influence of top-down expectations and bottom-up sensory input depends on their precision (i.e., uncertainty). In predictive coding architectures, this is implemented hierarchically: higher-level regions generate predictions about expected interoceptive input, while lower-level regions signal prediction errors that are used to update those predictions. Within this predictive and interoceptive framework, what has traditionally been termed core affect can be reinterpreted as the phenomenological expression of the organism’s ongoing regulatory loop. More specifically, valence (pleasant–unpleasant) indexes how supportive or costly the currently predicted bodily state is for the organism’s energy budget, while arousal (activated–quiescent) reflects the degree of mobilization being prepared in light of anticipated demands (Barrett, 2017).

Core affect is therefore continuously present because the brain is continuously engaged in regulating bodily resources. This aligns with Russell’s characterization of core affect as an “ever-present, background feeling state” organized along the dimensions of valence and arousal (Russell and Barrett, 1999; Russell, 2003), as well as with Cummins’s notion of a homeostatically protected baseline mood (HPMood) as an immediate, pre-conceptual affective tone (Cummins, 2016). From this perspective, emotional life is not a separate layer added onto regulation, but is inseparable from it: what we consciously feel — how pleasant or unpleasant, activated or calm we are — is the experiential readout of predictive body regulation unfolding in real time.

This account can be further specified computationally. Yanagisawa et al. (2019) propose a Bayesian model in which encounters with the environment perturb this regulatory loop by generating information gain — formally defined as the Kullback–Leibler divergence between prior and posterior beliefs. In their framework, information gain serves as a computational measure of surprise and predicts subjective arousal. Crucially, arousal is not determined solely by the magnitude of prediction error, but by its interaction with prior uncertainty. When prediction error is small, greater prior uncertainty leads to higher arousal; when prediction error is large, lower prior uncertainty amplifies arousal — a pattern they describe as the arousal crossover effect.

Within the same model, valence is conceptualized as the combined output of reward and aversion systems, each modeled as a sigmoidal function of information gain. Low information gain is associated with under-stimulation and negative valence; moderate information gain with positive valence; and very high information gain with overload and aversive experience. The resulting relationship follows an inverted U-shape, consistent with classical hedonic theories, although this formalization of valence remains primarily theoretical.

**FIGURE 2:**
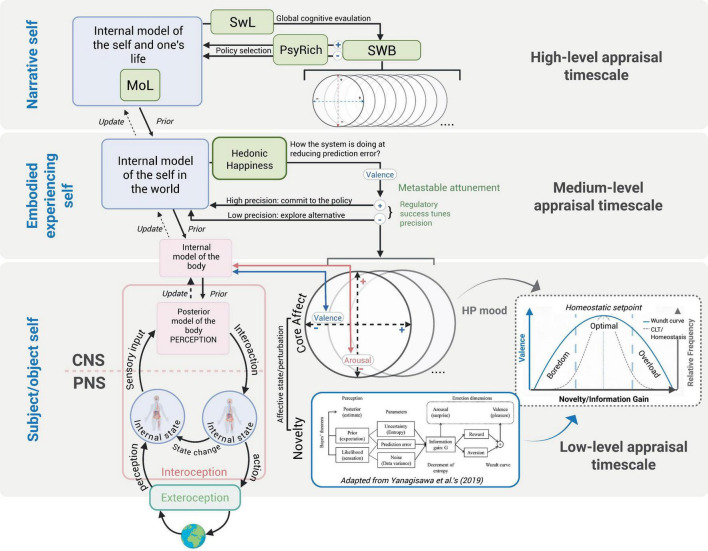
A hierarchical model of subjective well-being grounded in predictive processing and active inference, linking bodily regulation, affective dynamics, and higher-order evaluation across timescales. At the interoceptive (low) level, the organism regulates its internal states through a continuous perception–action loop involving central and peripheral systems. Here, core affect—defined by valence and arousal—reflects the ongoing predictive regulation of the body. Valence indexes how efficiently prediction errors are being reduced relative to expectations, while arousal reflects the degree of mobilization required to meet anticipated demands. Environmental changes are formalized as information gain (novelty), which drives Bayesian belief updating. Within this framework, arousal is represented by information gain, whereas valence is modeled as a non-linear function of information gain that depends on prior uncertainty (Yanagisawa et al., 2019). Core affect fluctuates within a homeostatically protected range (HPMood), conceptualized as a preferred region of affective states rather than a fixed point. This range corresponds to the optimal zone of the Wundt curve, where intermediate levels of stimulation are both metabolically efficient and experientially favorable. The tendency to occupy this region is further supported by the Central Limit Theorem (CLT), which predicts that most environmental and internal conditions cluster around moderate values. At the intermediate (policy/affective) level, the system tracks its recent dynamics. Hedonic happiness reflects a short- to medium-term integration of core affect, indicating how closely ongoing states align with the preferred range. At this level, valence also functions as a control signal by tuning precision: positive valence stabilizes current policies, whereas negative valence promotes exploration and updating. This supports metastable attunement, allowing flexible transitions between stability and change. At the higher (narrative/evaluative) level, subjective well-being (SWB) emerges as a longer-term appraisal integrating affective dynamics and cognitive evaluations, including Satisfaction with Life (SwL), Meaning in Life (MoL), and Psychological Richness (PsyRich). From a Bayesian perspective, SWB can be interpreted as a higher-order estimate of model evidence, reflecting how effectively the system maintains viable states over time relative to its expectations. These levels are hierarchically linked through reciprocal exchanges: higher levels provide top-down priors that shape expectations at lower levels (solid arrows), while lower-level signals supply bottom-up updates that revise higher-order beliefs (dashed arrows). At the neural level, this organization corresponds to systems of increasing integrative complexity, from interoceptive and visceromotor regions to large-scale networks supporting valuation, control, and narrative self-representation, following a scale-dependent organization. Overall, well-being is modeled as an emergent property of hierarchical predictive regulation, arising from the continuous interaction between homeostatic stabilization (maintaining preferred states) and allostatic adaptation (updating those states as conditions change).

The resulting inverted-U relationship is conceptually consistent with the classic Wundt curve, which describes an inverted U-shaped relationship between subjective pleasantness and stimulus intensity or arousal (Wundt, 1897/1998; Berlyne, 1971; Veldhuizen et al., 2006). Very low levels of stimulation are experienced as dull or disengaging, whereas very high levels are experienced as overwhelming or aversive. By contrast, intermediate levels are typically experienced as most pleasant and engaging (see Figure 2, low right panel). Berlyne (1971) extended this principle beyond sensory intensity to variables such as novelty, complexity, and uncertainty, suggesting that both monotony and chaos are aversive, while optimal experience lies within an intermediate range.

Interestingly, this preference for intermediate levels of information gain parallels the statistical argument advanced by Marks (2024). The Central Limit Theorem implies that when many independent sources of variation combine, their aggregate tends to cluster around a mean, producing a normal distribution (de Moivre, 1733/1967; Gauss, 1809; Lindeberg, 1922). Applied to organismic life, this suggests that most environmental and internal conditions — including levels of stimulation, metabolic demand, and social input — tend to fall within a moderate range, while extreme conditions are comparatively rare. If organisms are most frequently exposed to moderate conditions, then regulatory systems will be tuned to operate efficiently within this range. In this zone, predictions are more accurate, metabolic regulation is less costly, and behavior is more reliable. Moderate stimulation is therefore not only statistically typical but also metabolically optimal. This alignment between expected and encountered states may be precisely what is experienced as pleasantness or engagement — the peak of the Wundt curve.

By contrast, conditions at the extremes of the distribution impose greater regulatory costs. Under-stimulation deprives the system of meaningful input and is experienced as boredom or apathy, whereas over-stimulation demands high levels of compensatory mobilization and is experienced as stress or aversion. In this way, statistical typicality, metabolic efficiency, and hedonic preference converge: the conditions that are most common are also those that are easiest to regulate and, consequently, most likely to be experienced as subjectively optimal.

This alignment has direct implications for how subjective well-being can be understood theoretically. If most of life unfolds within a moderate, statistically likely regime — one that is both metabolically sustainable and hedonically optimal — then “feeling well” can be conceptualized as successfully remaining within this viable corridor, and returning to it when perturbed. This view resonates with the core intuition underlying homeostatic models of well-being, including Marks’s Homeostasis Theory (Marks, 2024) and Cummins’s account of Homeostatically Protected Mood (HPMood; Davern et al., 2007; Cummins, 2010).

Within this perspective, the “ever-present feeling state” described by Russell (2003) can be seen as anchoring everyday experience around such preferred ranges. In Bayesian terms, these ranges correspond to learned, precision-weighted preferred distributions — that is, expected states that the generative model actively seeks to occupy and maintain. These priors guide both perception and action, ensuring that the organism remains within metabolically and behaviorally sustainable conditions (Friston, 2010; Barrett and Simmons, 2015; Stephan et al., 2016; Parr and Friston, 2019; Petzschner et al., 2021). Concretely, the brain implements these preferences by generating predictions about desired interoceptive states and selecting actions that bring sensory input into alignment with those predictions, thereby minimizing expected free energy over time. The precision of these priors determines how strongly the system resists deviation from its preferred range.

At the same time, because environmental demands and bodily needs are continuously changing, these preferred states cannot remain fixed. Through allostatic processes, the system updates its priors — effectively recalibrating its set points — so that the attractor landscape within which regulation unfolds can shift, widen, or narrow while remaining viable. In this sense, well-being emerges from the dynamic interplay between homeostatic stabilization and allostatic adaptation.

Within Lisa Feldman Barrett’s constructionist framework, this regulatory process directly grounds emotional experience. Because the brain’s primary task is allostatic regulation, emotion episodes arise when learned emotion concepts are used to categorize moment-to-moment shifts in core affect within a given context, thereby selecting policies that regulate the body *in situ*. Variability is therefore expected, as multiple neural and bodily configurations can instantiate the same functional category (degeneracy).

Yanagisawa et al. provide a complementary mechanistic account of how such affective shifts arise. In their model, encounters with the environment generate information gain — that is, belief updating — which perturbs the regulatory loop and shifts core affect (see Figure 2). When integrated, these perspectives define a coherent pipeline: allostatic predictions set the body’s expected range; incoming evidence, weighted by precision, drives updates that shift core affect; and concept-based categorization transforms these shifts into emotion while guiding context-appropriate regulation (Barrett, 2017; Yanagisawa et al., 2019). This framework explains why the same situation may be experienced as engaging, boring, or overwhelming: the outcome depends on the interaction between prediction error, prior precision, and available conceptual models.

From this perspective, moment-to-moment fluctuations in information gain systematically tilt core affect toward engagement, boredom, or overload, while subsequent conceptualization shapes these shifts into emotion episodes. Subjective well-being, in turn, reflects the system’s capacity to keep these updates within an assimilable range — maintaining core affect near protected states while flexibly integrating perturbations. When this process functions effectively, novelty is experienced as learning rather than threat, and the organism remains within a metabolically sustainable and experientially optimal regime.

Taken together, the theoretical and computational elements discussed above can be integrated into a unified multilevel predictive model of well-being, schematically represented in Figure 2. The following sections further elaborate on its specific implications.

## A Bayesian theoretical account of health

Having clarified the proposed model of subjective well-being in predictive processing and active inference terms, the next step is to extend this framework to health. A useful entry point is the work of Miller et al. (2022), who reconceptualize mental health and psychopathology in terms of generative models, precision control, and metastable attunement across timescales.

In their Bayesian account, mental health depends on the adequacy of the organism’s generative model — that is, its capacity to capture the causal structure of the body–environment system and to guide adaptive action over time. A well-functioning model anticipates likely demands and selects policies that keep surprise bounded, thereby supporting both short-term physiological stability (homeostasis) and longer-term anticipatory adjustment (allostasis). In computational terms, this corresponds to maximizing model evidence, or minimizing expected surprise, in line with the “good regulator” principle (Conant and Ashby, 1970; Miller et al., 2022).

Affect plays a central role in this regulatory architecture. Valence reflects how the system is performing relative to its own expectations: positive affect signals that prediction error is being reduced more efficiently than expected, whereas negative affect indicates underperformance. In this sense, valence tracks the *direction and rate* of regulatory success and functions as a control signal. It modulates precision — the confidence assigned to beliefs and policies — thereby shaping the balance between exploitation and exploration. When a strategy is outperforming expectations, precision increases and the system commits to it; when performance declines, precision relaxes, allowing alternative policies to be explored. Through this loop, affect directly influences learning, action selection, and adaptation across time.

Mental health, then, is not simply a matter of minimizing error, but of allocating precision flexibly and context-sensitively. Psychopathology arises when this flexibility is lost — when precision becomes too rigid, too weak, or fixed where it should remain adjustable. Addiction illustrates this dynamic clearly. Repeated drug use leads the system to expect rapid reductions in prediction error from a narrow set of behaviors, causing precision to collapse onto a single policy. The agent becomes highly confident in one course of action, while alternative, longer-term strategies lose influence. What emerges is a self-reinforcing but suboptimal attractor: locally rewarding, yet globally constraining.

At this point, Canguilhem’s notion of health offers a crucial reframing. For Canguilhem (1966), health is fundamentally a matter of normativity: the organism’s capacity to establish, sustain, and transform its own norms of existence in relation to a changing environment. Health is not equilibrium, nor mere statistical normality, but the ability to generate new ways of living when existing ones no longer suffice. Illness, conversely, is a reduction of this normative capacity — a narrowing of the range of viable relations with the world (Vissio, 2020; Mauro et al., 2025a).

Within a Bayesian framework, this capacity can be understood as emerging from the interaction of priors, precision-weighting, and policy selection mechanisms. However, rather than being reducible to priors alone, normativity concerns how the system actively negotiates what counts as viable, valuable, and worth pursuing across timescales. In this sense, it is expressed in the ongoing organization and reorganization of policies under both homeostatic and allostatic constraints. At lower levels, normativity is tightly constrained by interoceptive and allostatic demands, which impose strong boundaries on acceptable states and limit the space of viable policies. At higher levels, it manifests as the capacity to expand, revise, and recombine policies in light of new information, goals, and environmental affordances. Normativity is therefore not a static set of preferred states, but a dynamic property of the system’s ability to generate new norms—that is, new viable ways of acting and engaging with the world.

From this standpoint, the “bad bootstrap” described by Miller and colleagues can be understood as a form of normative rigidity. The system is not devoid of norms; rather, it is locked into a narrow and inflexible one. Precision becomes concentrated on a limited set of policies, while the capacity to reorganize behavior around new meanings, goals, or relationships is diminished. Psychopathology, in this sense, is not only a failure of inference, but a loss of normative flexibility — a reduced ability to redefine what counts as viable or meaningful engagement with the world. This perspective also clarifies the notion of sustained well-being. Rather than constant pleasure or minimal surprise, well-being is better understood as the result of metastable attunement: a dynamic condition in which the system remains poised between stability and reorganization. It can settle into coordinated patterns of action, affect, and meaning, yet also revise them when conditions change. Valence-guided precision tuning enables this balance: positive affect stabilizes effective patterns, while negative affect destabilizes them, opening space for reconfiguration. This mechanism supports resilience, learning, and the capacity to shift across contexts and timescales.

However, this account remains incomplete without considering the bodily, interoceptive dimension of experience — what has been described as core affect, the continuously accessible background state defined by valence and arousal (Russell, 2003, 2009; Cummins, 2010). As discussed in the previous section, valence indexes the predicted metabolic advantage or cost of the current bodily state, while arousal reflects the degree of mobilization required to meet anticipated demands (Barrett, 2017; Allen and Tsakiris, 2018).

This lower-level constraint is not optional. Because survival depends on maintaining bodily viability, allostatic predictions tend to carry high precision and strongly shape behavior. When interoceptive conditions are stable — that is, when core affect remains within a tolerable, homeostatically protected range — precision can be redistributed toward higher-order goals, allowing identity, values, and long-term projects to guide action. When bodily stability is threatened, however, precision collapses onto short-term regulatory imperatives, narrowing the system’s effective horizon.

A coherent integration follows from this. The framework of Miller and colleagues explains how valence-guided precision tuning supports adaptive behavior across timescales, while predictive allostasis clarifies why certain constraints — especially those related to bodily regulation — take priority. Core affect provides the phenomenological signature of these constraints, indicating whether the system has sufficient stability to explore or must return to immediate regulation. When the organism is sufficiently regulated, its normative range expands; when it is not, that range contracts.

On this basis, happiness can be understood as the felt signal that ongoing regulation is proceeding better than expected at an acceptable energetic cost. It is more extended than momentary affect, yet remains a relatively proximal appraisal of regulatory success rather than a higher-order evaluation of meaning, identity, or normative coherence across time, a distinction that will be further specified in the discussion that follows.

## A Bayesian theoretical account of happiness

Looking at these more extended timescales, it is useful to consider that in contemporary psychology, models of happiness and psychological well-being—understood as theories of what constitutes a “good life”—offer powerful insights for explaining how positive emotion and life satisfaction scaffold resilience, adaptive coping, and health-promoting behavior (Mauro et al., 2025a; Ryff and Keyes, 1995; Fredrickson, 2001). The classical account distinguishes a hedonic component (happiness) from a eudaimonic component (meaning and purpose; Ryff, 1995; Ryff and Singer, 1998) of good life, while a recent extension introduces a third, complementary strand—psychological richness—defined as experiencing life as varied, interesting, and perspective-shifting (Oishi and Westgate, 2022). Although psychological richness correlates with happiness and meaning, it has distinct antecedents and consequences, suggesting a unique contribution to judgments of a life well lived (Oishi and Westgate, 2022; Mauro et al., 2025b).

From a Bayesian/active-inference standpoint, this tripartite model of well-being—hedonic happiness, eudaimonia (meaning), and psychological richness—maps onto complementary components of a generative agent: online affective evaluation (valence as a performance signal), slow identity-level priors (values and purposes), and likelihood-seeking exploration (informative sampling). More specifically, adopting this perspective, as suggested by Miller et al. (2022), happiness may represent the felt signal that one’s current and anticipated regulation of self and world is working: prediction errors are being reduced in line with one’s needs and values, at acceptable energetic cost, and the future looks controllable enough that the organism can safely invest in it (Barrett, 2017; Stephan et al., 2016; Van de Cruys, 2017; Miller et al., 2022; Smith et al., 2022), while meaning in life may represent instead the felt alignment between one’s present actions and higher-order priors about identity, values, and desired futures (Smith et al., 2022). At a higher level, it signals that current behavior not only regulates immediate demands but also reduces expected future surprise at the level of “who I am” and “what my life is for” (Friston, 2010; Pezzulo et al., 2015). Hence meaning can coexist with effort and suffering: short-term costs are tolerated when actions stabilize identity-relevant predictions (e.g., “I am a good parent,” “I defend what matters”) (Stephan et al., 2016; Miller et al., 2022). Formally, the system minimizes not only momentary physiological prediction error (core affect) but also long-horizon, self-relevant error—the gap between the person expected and the person becoming through action (Smith et al., 2022; Stephan et al., 2016). Loss of meaning is otherwise destabilizing because it collapses high-level priors, leaving day-to-day regulation intact but undermining sustained, goal-directed control (Stephan et al., 2016; Miller et al., 2022). Thus, “meaning” is not only a metaphysical answer but also an ongoing control process that maintains a coherent, credible narrative in which present actions count as progress toward an endorsed future (Friston, 2010; Smith et al., 2022).

As discussed, from an active inference perspective, an organism constantly maintains an internal model that predicts what states it expects to occupy, what sensations it is likely to encounter, and which actions will keep it within viable and valued states over time (Friston, 2010; Pezzulo et al., 2015). Typically, the system tries to minimize surprise by reducing prediction error and keeping its internal milieu within tolerable bounds — physiologically, socially, emotionally (Barrett et al., 2016; Stephan et al., 2016). Chronic uncontrollable uncertainty, where the environment is volatile and prediction errors cannot be resolved, is associated with anxiety, helplessness, and depressive collapse, because the agent learns “I cannot regulate myself or my world” (Stephan et al., 2016; Clark, 2013; Kiverstein et al., 2020).

Psychological richness can be seen as a different style of regulation: instead of treating all uncertainty as threat, the agent deliberately inhabits and explores certain kinds of uncertainty, using them to expand and refine its own model. From this perspective, psychological richness is a life structured to generate repeated, meaningful updates to one’s internal model — encounters, contexts, ideas, and emotional situations that force the agent to revise who it thinks it is and what it thinks the world is like (Oishi and Westgate, 2022; Miller et al., 2022). These are not chaotic, traumatizing shocks, but “informationally dense” experiences that produce assimilable surprise: cultural dislocation, deep conversation, intense art, awe, or even safe confrontation with fear. Under active inference, such experiences drive learning because they generate prediction errors that can still be integrated, rather than prediction errors that only overwhelm (Andersen et al., 2023; Deterding et al., 2022).

Formally, the trade-off between adaptive exploration and maladaptive volatility is regulated by the precision-weighting of prediction errors, their magnitude (information gain), and allostatic constraints on bodily viability, which together determine whether uncertainty is assimilable and learning-promoting or destabilizing and aversive. Seen this way, psychological richness corresponds to an expansion of one’s preferred state space (defined by the CLT/Wundt curve).

In active inference, agents act to keep themselves within “expected” or “preferred” states – ranges of bodily, emotional, and social conditions that feel viable and self-consistent (Friston, 2010; Smith et al., 2022). However, psychological richness should not be understood as a preference for specific states per se, but as a higher-order prior over epistemic policies, assigning value to information gain and enabling the expansion of the agent’s preferred state space.

Accordingly, a psychologically rich life is one in which those preferred states become broader, more flexible, and less fragile. The person learns to tolerate — and eventually endorse — a wider range of feelings, perspectives, roles, and situations without interpreting them as existential threat. In other words, they can remain regulated, coherent, and agentic in states that would previously have been coded as “too foreign” or “too destabilizing.” This widening of tolerable self-states is directly relevant to flourishing because it prevents the system from becoming rigid, hyper-defensive, and brittle (Miller et al., 2022; Carhart-Harris and Friston, 2019; Laukkonen and Slagter, 2021; Mauro et al., 2025b).

Psychological richness also reflects a trained stance toward uncertainty. Under ordinary stress, agents tend to assign very high precision (very high weight) to threat-related prediction errors, which forces defensive, narrowing behaviors — avoidance, withdrawal, compulsive control (Barrett et al., 2016; Stephan et al., 2016). In contrast, a psychologically rich stance looks more like what has been described in terms of play and exploratory engagement: the person learns that some uncertainty is workable, even valuable, and can be entered without immediate shutdown (Andersen et al., 2023; Miller et al., 2022). This is similar to “consumable error,” where the agent seeks challenges at the edge of its current ability — enough novelty to generate prediction error, but not so much that regulation fails — and experiences positive affect when it successfully reduces that error faster or more effectively than expected (Van de Cruys, 2017; Kiverstein et al., 2019; Deterding et al., 2022). Over time, this reinforces the higher-order prior: “I can enter the unknown and adapt.” And that prior is itself protective.

Finally, psychological richness can be seen as epistemic resilience. A system that only ever minimizes immediate surprise risks overfitting its world, becomes narrow, rigid, addicted to sameness, and vulnerable to shock when something truly unexpected happens (Carhart-Harris and Friston, 2019). A psychologically rich life continuously exposes the system to alternative models of reality — alternative meanings, identities, moral frames, social worlds — in ways that keep its generative model plastic rather than frozen (Oishi and Westgate, 2022; Miller et al., 2022). In Bayesian terms, this reduces catastrophic prediction error later, because the agent has already rehearsed, integrated, and metabolized multiple possible ways the world (and the self) can be. Interestingly enough, if the construct of psychological richness describes a life dense with perspectives—varied, complex, sometimes bittersweet experiences that change how one sees oneself and the world, playfulness is one of the stances that makes uncertainty explorable, turning routines into small discoveries and difficulties into experiments in meaning.

As a matter of fact, within active inference framework, Kiverstein and Miller (2023) argue that playfulness is a core ingredient of a meaningful, flourishing life, framing it as a neurocomputational process that supports meaningful living (Ryan and Deci, 2001; Seligman, 2011; Diener et al., 1985). According to this view, uncertainty is therefore not uniformly harmful: while chronic, volatile uncertainty undermines perceived control and allostatic self-efficacy (Barrett et al., 2016; Stephan et al., 2016; Paulus et al., 2019), a playful stance installs a high-level prior that treats selected uncertainty and prediction error as welcomed, explorable, and regulable, widening one’s tolerated surprise range and contributing to both momentary enjoyment and long-term meaning (Andersen et al., 2023; Miller et al., 2022).

From this stance follow three interlocking effects that align playfulness with psychological richness. First, playfulness supports an open, present-centered attention that is less dominated by rigid habits and self-focused filtering, enabling receptive engagement with the here-and-now (Bar, 2022). Second, it keeps agents at the edge of ability, generating “consumable errors” (novel yet assimilable) whose resolution yields learning progress and positive valence—consistent with accounts that tie affect to doing better-than-expected at reducing meaningful prediction errors—and, over time, scaffolds skills, self-efficacy, and growth (Andersen et al., 2023; Deterding et al., 2022; Van de Cruys, 2017; Kiverstein et al., 2019; Vygotsky, 1978; Kashdan and Steger, 2007; Seligman, 2011). Third, playfulness fosters metacognitive insight into effect: safe simulations (e.g., watching horror movies, intense competition) function as laboratories for precision redeployment, so signals like fear or craving are treated as informative but optional, increasing flexibility and resilience (Sandved-Smith et al., 2021; Scrivner, 2021a,b; Bowen and Marlatt, 2009; Miller et al., 2022).

Summarizing, flourishing in the active inference framework is not mere local error minimization but also minimizing expected future error at identity-relevant levels even when this entails short-term effort and stress to align action with higher-order purposes (Stephan et al., 2016; Miller et al., 2022). Playfulness thus emerges not as trivial pleasure but as a regulatory style (trait-level prior) that treats selected uncertainty as workable, preventing prior rigidification and enabling openness, creativity, curiosity, growth, and a metacognitively informed relationship to affect—potentially a key computational ingredient of a meaningful life (Carhart-Harris and Friston, 2019; Laukkonen and Slagter, 2021). This framing also aligns with recent work suggesting that psychological richness—beyond pleasure and meaning—constitutes an important dimension of happiness in its own right (Oishi and Westgate, 2022). From an active inference perspective, a psychologically rich life is one that remains open to complex, novel, and perspective-shifting experiences that may not maximize immediate comfort, yet expand the agent’s generative model, diversify the repertoire of viable policies, and increase long-horizon flexibility. In this sense, playfulness can be seen as a trait-like facilitator of psychological richness: by treating selected uncertainty as workable, it helps sustain epistemic foraging, supports identity-level updating, and allows happiness to be grounded not only in feeling good or living meaningfully, but also in the felt depth and texture that come from engaging with a broader range of experiences (Oishi and Westgate, 2022).

## Cultivating well-being: mindfulness and psychotherapy in a Bayesian perspective

As we saw in the previous sections, cultivating well-being requires more than illness or symptom relief; it involves stabilizing mood, enriching meaning, and sustaining flexible, values-congruent action in daily life. Two evidence-based routes—mindfulness cultivation and psychotherapy—may work synergistically toward these aims.

### Mindfulness

Mindfulness—often defined as intentional, non-judgmental attention to present-moment experience—has showed reliable benefits across mental health and well-being. Randomized trials and meta-analyses link mindfulness training to reductions in anxiety, depression, and stress, alongside gains in life satisfaction and self-compassion (Hofmann et al., 2010; Goyal et al., 2014; Khoury et al., 2013). Standardized programs such as MBSR and MBCT improve symptom burden and, in recurrent depression, reduce relapse risk while enhancing quality of life (Kabat-Zinn, 2003; Kuyken et al., 2015; Segal et al., 2012). From the cognitive point of view, mindfulness strengthens attentional control and decentering, recalibrates interoceptive awareness, and dampens maladaptive habitual reactivity. Such changes are supported by neuroplastic effects in networks for self-regulation and salience (Garland et al., 2015; Lindsay and Creswell, 2017; Tang et al., 2015). In short, by shaping how one attends, appraises, and responds, mindfulness fosters both symptom relief and eudaimonic growth.

“*Zen Mind, Beginner’s Mind*” is a famous mindful core practice-centered invitation to cultivate “beginner’s mind” through meditation—an attitude of open, unprejudiced attention in which possibilities are many and certainty is few (Suzuki, 2020). In his namesake book, Suzuki places sitting meditation (Zazen) at the heart of Zen tradition, like many meditative traditions do, included the secular ones: an upright posture, natural breathing, and non-grasping awareness. In this context, practice is not a technique to secure some kind of enlightenment (i.e., cultivation of well-being, present awareness) later; it is itself the expression of enlightenment in the present moment. Recurrent themes include the “no gaining idea” (dropping self-improvement grasping), wholehearted effort without strain, letting thoughts arise and pass, and trusting simple forms of action—sitting, walking, breathing—as the way reality clarifies itself.

Viewed through active inference, the beginner’s mind attitude is a regime of precision control: it down-weights overconfident high-level priors while up-weighting trustworthy sensory and interoceptive likelihoods, keeping the agent curious, flexible, and resistant to the premature stabilization of high-level beliefs in the face of limited evidence. In line with this account, recent theoretical work suggests that meditation can be understood as systematically modulating the precision of hierarchical priors, particularly those related to self-models, thereby increasing sensitivity to incoming sensory and interoceptive signals and enhancing model flexibility (Laukkonen and Slagter, 2021). Rather than eliminating predictive structure, this process relaxes rigid top-down constraints, allowing experience to update the generative model more directly. The meditation practice provides the scaffold: the simple posture–breath routine stabilizes proprioceptive and interoceptive predictions, reduces uncontrolled autonomic volatility, and turns moment-to-moment sensations into clean, consumable prediction errors rather than possible threats to be defended against. The felt ordinariness yet clarity of practice follows: at its root mindfulness reduces free energy not by adding concepts, if not those which lead to improve the signal-to-noise ratio of experience, allowing the world to update the priors directly.

As a matter of fact, previous studies have showed that interoceptive awareness (IA) and dispositional mindfulness form two coordinated constellations—regulatory awareness and acceptance-in-action— strongly interrelated, and that each uniquely predicts psychological well-being (Hanley et al., 2017). From the active inference perspective, the unique predictive contributions of IA and mindfulness to well-being imply partially independent levers in the hierarchy: IA tunes the sensory likelihood and low-level precision (how bodily evidence is weighted), while dispositional mindfulness adjusts higher-level priors and precision control policies (when and where confidence is allocated). Moreover, it has been argued that many benefits attributed to mindfulness are better explained through interoception and its neural bases—especially the insula and the broader interoceptive network (Gibson, 2019). As we saw in the previous sections, interoception may be considered a multidimensional construct encompassing both perceptual and evaluative/top-down components. Mindfulness-related interventions tend to enhance subjective dimensions of interoception and are associated with insular neuroplasticity, whereas results on objective accuracy measures (such as heartbeat detection) seem to be inconsistent; thus, well-being appears more tightly linked to subjective/regulatory facets than to raw perceptual accuracy (Gibson, 2019). This pattern implies although there are different ways of how in meditation attention is directed to the body (e.g., focused attention meditation, open monitoring meditation, self-compassion, non-dual awareness), in all cases, the body serves as a platform for self-regulation, meta-awareness, and decentering.

Well-being, in this picture, reflects the long-horizon tendency of the system to minimize expected free energy, where IA and mindfulness are “tightly interwoven, partly overlapping,” yet each uniquely predicts flourishing—maps neatly onto distinct but coupled Bayesian instances: calibrate the likelihoods from the body and cultivate meta-priors that keep precision flexible. In this sense, a recent work from Van Gordon et al. (2023), argues that modern pursuits of happiness often backfire and that a contemplative reframing—grounded in non-attachment and mindful observation—offers a more stable route to well-being. Despite rising global wealth, population happiness has declined in many places, suggesting that how we seek happiness matters as much as external conditions. The authors frame happiness as one transient expression within a broader “ocean” of emotional energy, cautioning that rigid definitions narrow perception and fuel grasping; like waves, emotions arise and subside from the same underlying mind–brain processes. Treating happiness as a goal to possess sets up a roller coaster of pursuit, brief attainment, and loss, which can even foster self-criticism or fear of positive emotion.

Drawing on Buddhist sources, they advocate non-attached existence: relish each moment without clinging, cultivate “right view,” thus moving beyond accepting and rejecting. By seeing impermanence directly, one stops fighting to secure lasting highs or to annihilate lows. This stance reframes happiness and suffering as two sides of the same coin, both textures of consciousness rather than opposing absolutes.

Read through active inference, the “pursuit of happiness” problem is a case of maladaptive precision and policy selection. When an agent sets an over-precise prior on a narrow hedonic target—“I must feel happy and keep it”—the prior over policies collapses onto actions that promise that state. Because the world is volatile, sensory evidence frequently violates this prior, generating large affective prediction-error spikes and driving defensive control policies (grasping, suppression, rumination). The roller-coaster the authors describe—pursuit, brief attainment, loss—emerges as oscillations in policy precision: high confidence during pursuit, sharp error at mismatch, and compensatory tightening that further narrows future choices. In this context non-attachment functions as precision meta-control. By relaxing confidence in specific hedonic outcomes and increasing tolerance for uncertainty, the agent broadens the posterior over acceptable states and keeps policy precision tempered, so moment-to-moment variations in core affect become consumable errors rather than threats. Mindful observation clarifies likelihoods: sustained, non-reactive sampling of interoceptive and exteroceptive signals improves the mapping between cues and causes, raising the signal-to-noise ratio without inflating top-down demands. The “ocean and waves” metaphor (Van Gordon et al., 2023), fits a hierarchical model in which low-level affective fluctuations (waves) are expected under higher-level beliefs that include impermanence (the ocean); holding concepts lightly prevents categorical overfitting (“this must be happiness/sadness”) and reduces binary, all-or-nothing policy triggers. On this account, well-being improves not because highs are secured and lows eliminated, but because the system minimizes long-horizon expected free energy: fewer spurious alarm precisions, steadier learning at the edge of competence, and actions that remain flexible and context-sensitive even as affect rises and falls—happiness and suffering as two workable textures of the same inferential stream.

Importantly, although mindfulness and meditation practices are often associated with improved well-being, a growing body of research indicates that they may also give rise to adverse or destabilizing experiences, including anxiety, dysregulated arousal, depersonalization, and altered sense of self (e.g., Schlosser et al., 2019; Farias et al., 2020). Within a predictive processing framework, these effects can be understood as a consequence of the relaxation of high-level priors—particularly those related to self-models—without sufficient integration at other levels of the hierarchy.

While such relaxation may enhance sensitivity to incoming information and promote learning under appropriate conditions, it may also increase exposure to unfiltered prediction error. When this influx of information exceeds the system’s capacity for integration—due to insufficient precision regulation, lack of conceptual scaffolding, or limited affect tolerance (e.g., because of trauma; Siegel, 1999) —experience may shift from epistemic exploration to maladaptive volatility.

In this sense, the distinction between adaptive and maladaptive effects of meditation can be framed in terms of whether prediction errors remain assimilable within the generative model.

From this perspective, psychotherapy—and in particular the Referential Process (Bucci, 1997)—may play a crucial complementary role. By facilitating the translation of subsymbolic and affective experience into symbolic and narrative forms, psychotherapy supports the integration of otherwise destabilizing prediction errors into coherent representations. This process effectively restores cross-level coupling within the generative hierarchy, enabling the system to expand its model without losing stability.

**FIGURE 3:**
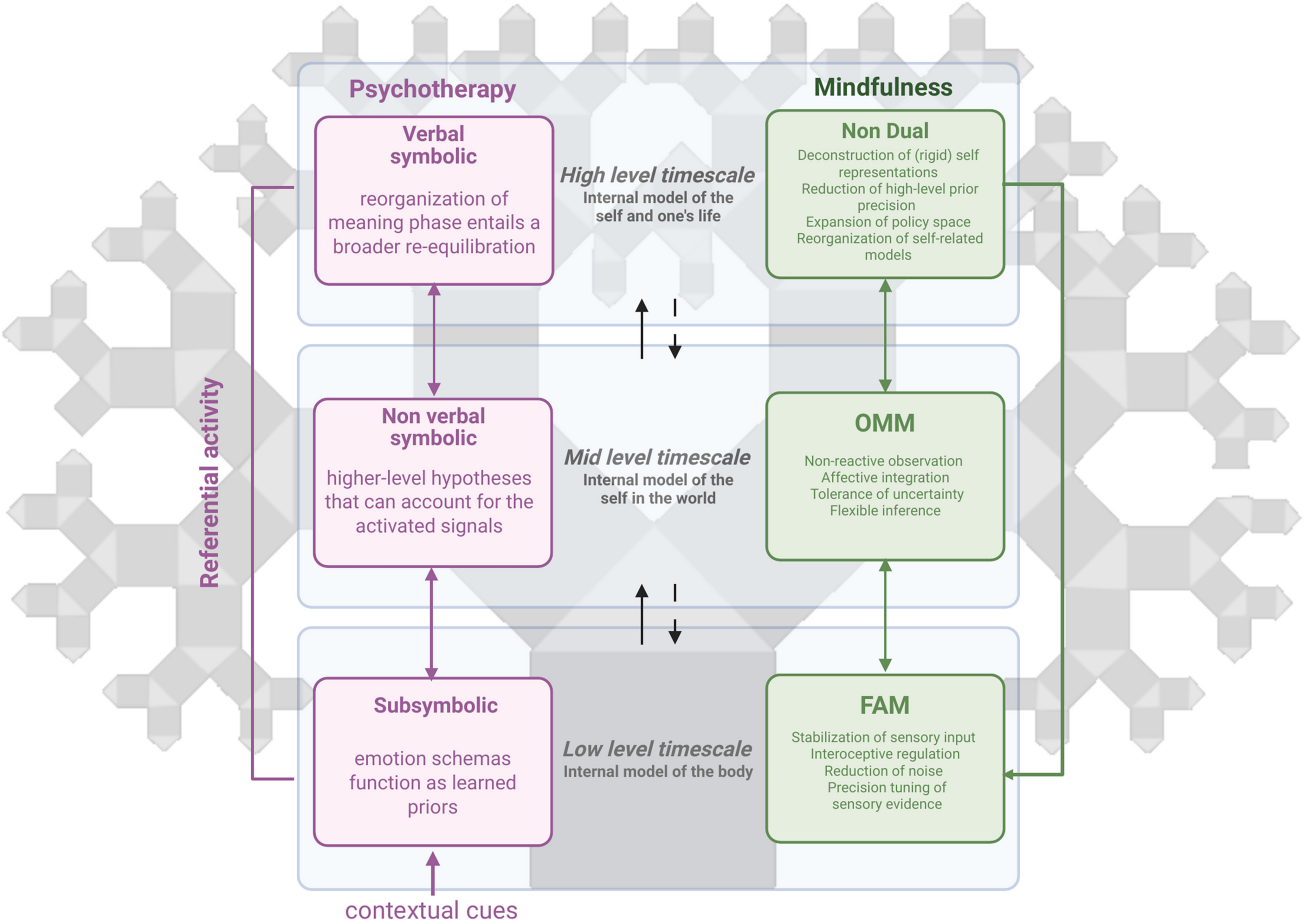
A multilevel predictive model integrating psychotherapy and mindfulness practices within an active inference framework. Three hierarchical levels are depicted, corresponding to distinct timescales and forms of representation: interoceptive, embodied, and verbal–narrative. The modules represented on the right correspond to established mindfulness practices: Focused Attention Meditation, which involves sustaining attention on a selected object (e.g., the breath) and redirecting it when distracted; Open Monitoring Meditation, characterized by a non-reactive awareness of ongoing experience without focusing on a single object; and Non-dual awareness, which entails a shift from object-based attention toward recognizing awareness itself, with a reduced distinction between observer and observed (Lutz et al., 2008; Jha et al., 2007; Josipovic, 2014). Those on the left reflect the Referential Process within psychotherapy, encompassing the successive phases of emotional activation, symbolization (through images and language), and reflective reorganization of meaning. The hierarchical structure visually suggested by the Pythagoras tree in the background serves to map the functional areas of intervention within the model. In this sense, the figure represents the primary targets of regulation and transformation at each level, illustrating how different practices and processes modulate precision-weighting, belief updating, and policy selection across the hierarchy. In the case of mindfulness practices, this mapping refers to the areas related to the object of observation and phenomenological focus characteristic of each practice, rather than to the specific neural systems exclusively engaged by that form of meditation. While this sequential translation across representational levels is central to the Referential Process, in mindfulness subsymbolic experience — particularly in non-dual awareness — remains directly accessible, yet is no longer structured or constrained by higher-level representational priors.

Thus, rather than representing alternative or competing approaches, meditation and psychotherapy can be understood as operating on complementary aspects of the same regulatory architecture: meditation loosens rigid priors and increases openness to experience, while psychotherapy scaffolds the integration of that experience, ensuring that epistemic expansion remains within viable and adaptive bounds (see Figure 3).

### Psychotherapy

Against the broader picture that has emerged so far, fostering well-being appears less about eliminating aversive states than about reshaping the inferential ecology that renders them overwhelming. From this perspective, psychodynamic psychotherapy may be understood as one pathway through which such reshaping is achieved, by bringing implicit patterns—affect, defenses, and relational expectations—into awareness and rendering them more malleable. Through the therapeutic relationship—within a reflective and emotionally engaged context—patients develop insight, increase affect tolerance, and enhance reflective functioning and attachment security, with gains that extend beyond symptom relief to life satisfaction, role functioning, and relational quality. Evidence syntheses indicate that psychodynamic treatments are effective for common mental disorders with outcomes comparable to other bona fide therapies, and that gains often endure or continue to accrue after treatment—suggestive of deeper structural change (Shedler, 2010; Leichsenring and Klein, 2014; Driessen et al., 2015; Steinert et al., 2017). Long-term or intensive formats appear especially helpful for chronic, comorbid, and personality-related conditions (Leichsenring and Rabung, 2008; Abbass et al., 2014).

Positioned within the broader evidence base, psychodynamic psychotherapy is one among several validated approaches—including cognitive-behavioral, interpersonal, systemic, acceptance- and mindfulness-based, and humanistic therapies—with meta-analytic support for depression, anxiety, somatic-symptom, and personality disorders; here again, longer or more intensive formats show particular benefit for complex, enduring presentations (Shedler, 2010; Leichsenring and Klein, 2014; Driessen et al., 2015; Steinert et al., 2017; Leichsenring and Rabung, 2008; Abbass et al., 2014). Across modalities, psychotherapy reliably improves subjective well-being and quality of life, not only symptoms, with medium average effects and durable follow-up outcomes; common factors (e.g., alliance, goal consensus) explain much of this benefit, and specific methods refine it (Cuijpers et al., 2021; Bolier et al., 2013; Wampold and Imel, 2015; Hofmann et al., 2012).

Recent theoretical work has further suggested that the therapeutic action of both psychotherapy and mindfulness depends on cultivating curiosity toward ongoing experience. Rather than attempting to eliminate distress or simply reduce prediction error, curiosity allows patients to approach thoughts, emotions, and bodily sensations with an open and non-judgmental attitude, thereby facilitating insight into habitual predictive patterns. From an active inference perspective, this process supports the adaptive revision of maladaptive priors by enabling sustained engagement with prediction errors instead of defensive avoidance. In this view, therapeutic change emerges through a gradual increase in epistemic openness, allowing previously rigid generative models to become more flexible and better attuned to ongoing experience (Bowen and Marlatt, 2009).

To better understand how specifically psychodynamic psychotherapy supports psychological well-being, it may be useful to consider Wilma Bucci’s Multiple Code Theory (MCT) and Referential Process (RP)—a process model of how psychotherapy transforms emotion into words and meaning (Bucci, 1997). The Multiple Code Theory is a translational, cross-paradigm framework for understanding how emotion becomes thought and language in many clinical and research settings. It proposes that emotional information is represented and processed across three interconnected formats: a verbal symbolic system, a nonverbal symbolic system (e.g., discrete images, action plans), and a nonverbal subsymbolic system (e.g., continuous somatic/visceral patterns and sensory-motor microprocesses). Emotions are conceived as “emotion schemas”—image–action constellations that integrate sensory, motor, and visceral components and may operate inside or outside awareness (Bucci, 1997).

In this model (see Figure 3, left diagram), pathology arises when links among the three codifications of the experience are weak or developmentally underbuilt (Bucci, 1997); framed normatively, it reflects norm narrowing—reduced flexibility in establishing and re-establishing viable physiological, affective, and relational norms through their integration. As the repertoire of images, words, and actions is limited, experience is funneled through a few overlearned patterns: subsymbolic arousal is poorly symbolized, concepts become rigid, and behavior repeats in narrow loops. The result is reduced tolerance for variability and context, compromised regulation, and symptoms that reflect a system stuck enforcing too few “acceptable” ways of feeling, thinking, and relating. In this context somatization’s symptoms are reframed as transitional symbols that can organize otherwise unlinked subsymbolic arousal when interpersonal objects or words are warded off. Therapeutic change then depends on linking the three codes via the RP, a cyclic sequence of (1) arousal/activation of emotion schemas, (2) symbolizing/narrating experience in images and words, and (3) reflection/reorganization of meaning. Read through an active inference framework, Bucci’s three codes map onto levels of a hierarchical generative model, and the Referential Process (RP) is the message-passing cycle that aligns them by minimizing (expected) free energy over time. First, the arousal or activation phase can be understood as the emergence of bottom-up, precision- weighted prediction error from subsymbolic, interoceptive, and sensorimotor channels. At this level, emotion schemas function as learned priors concerning domains such as threat and safety or attachment and loss. When contextual cues—including the therapist’s interventions—enhance precision on these channels, latent affective states become salient and call for interpretation. Clinically, this corresponds to the felt surge of experience—somatic markers, imagery fragments, and action tendencies—that signals a mismatch between ongoing predictions and current input. Second, the symbolization and narration phase corresponds to model updating. Here, nonverbal symbolic (e.g., images, metaphors) and verbal symbolic (e.g., narratives) representations provide higher-level hypotheses that can account for the activated signals. Inference proceeds both by reshaping the generative model—introducing new latent causes and reweighting priors—and by selecting policies that sample evidence consistent with these emerging interpretations. In this sense, symbolization binds subsymbolic signals into shared representational formats, reducing uncertainty while enabling action. Third, the reflection and reorganization of meaning phase entails a broader re-equilibration. The system evaluates longer-horizon expected free energy, prunes maladaptive priors, and redistributes precision across levels. Narratives that better anticipate future affective and interpersonal outcomes are retained, while less adaptive ones are discarded. Clinically, this process manifests as increased differentiation (e.g., recognizing distinct emotional components), integration (linking bodily states, images, and language), and flexible revision of action policies. As precision shifts—away from rigid defensive expectations toward more nuanced appraisals—the system enters subsequent cycles from a more adaptive baseline.

Taken together, therapeutic change can be described as iterative active inference over emotion. Activation surfaces informative prediction errors; symbolization constructs candidate explanations and action policies; reflection consolidates these updates by optimizing model evidence over longer timescales. Across repeated cycles, subsymbolic, nonverbal, and verbal representations become more tightly coupled, prediction errors decrease in frequency and intensity, and behavior shifts from habitual avoidance toward more exploratory and value-consistent engagement. Within this framework, Barrett’s Theory of Constructed Emotion can be read as a complementary account of how affective dynamics are organized into meaningful experience. Emotions are constructed through the application of learned concepts to fluctuations in core affect—valence and arousal—in the service of adaptive regulation (Barrett, 2017). From this perspective, core affect aligns with subsymbolic processing, while emotion concepts map onto symbolic representations. Emotional episodes thus emerge when subsymbolic signals are successfully integrated into symbolic meaning, a process that closely parallels the Referential Process, whereby bodily and affective states are progressively translated into images and narratives that guide interpretation and action.

This convergence also clarifies the role of variability in emotional experience. In Barrett’s account, emotional categories exhibit *degeneracy*: the same emotion can be realized through multiple neural and bodily configurations depending on context and history (Barrett, 2017). This resonates with Bucci’s view that different coding pathways can yield functionally equivalent meanings (Bucci, 1997). Metastability provides the missing link between these perspectives, explaining how such variability remains adaptive: rather than settling into fixed patterns, the system operates near a range of possible configurations, allowing it to flexibly assemble and reorganize couplings between affect, imagery, and language as conditions change (Kelso, 2012; Bruineberg et al., 2021).

Within this context, psychotherapy can be understood as supporting this metastable attunement. By providing a relational environment in which precision is co-regulated, it allows rigid, overconfident priors—particularly those associated with threat or defensive responding—to soften, while enhancing the salience and reliability of embodied signals. This, in turn, enables the exploration of alternative interpretations and action tendencies without overwhelming the system, effectively expanding the repertoire of viable meanings and responses. In sum, if core affect provides the ongoing background for appraisal (Russell, 2003), well-being can be understood as the system’s capacity to flexibly maintain and revise its own viable states over time. Within this framework, psychotherapy supports this process by stabilizing experience while enhancing integrative flexibility, enabling bodily signals to be reinterpreted in multiple adaptive ways and thereby fostering epistemic resilience. Complementarily, mindfulness practices recalibrate precision by cultivating a non-judgmental awareness of interoceptive and exteroceptive signals, stabilizing visceromotor predictions and expanding the available policy space. In particular, non-attachment functions as a form of precision meta-control, relaxing overly rigid expectations about specific affective outcomes, increasing tolerance for uncertainty, and broadening the range of acceptable states. As a result, fluctuations in core affect become manageable rather than threatening, supporting flexible updating and context-sensitive engagement.

## Conclusion

In conclusion, the present article has proposed a Bayesian active-inference framework in which subjective well-being is best understood as the phenomenological readout of how effectively a prediction-driven organism regulates itself within viable bounds across nested biological, psychological, and social timescales. Within this perspective, core affect functions as a continuously available index of regulatory dynamics, reflecting—but not exhaustively determining—system performance. At the event level, valence and arousal can be interpreted as control-relevant variables with information-theoretic significance, shaping learning, action selection, and resource allocation through precision-weighted prediction error.

Extending this framework to health, well-being is grounded in a regime of metastable attunement, in which stability is maintained while preserving the capacity for adaptive updating and reorganization. In line with Canguilhem’s notion of normativity, health is not reducible to equilibrium, but reflects the organism’s capacity to establish, sustain, and transform its own norms of existence in relation to a changing environment. Within a hierarchical Bayesian architecture, this capacity is expressed in the flexible organization of policies across timescales, allowing the system not only to maintain viable states but also to revise what counts as viable. Within this inferential hierarchy, distinct dimensions of well-being can be differentiated according to temporal horizon and representational depth. Hedonic happiness corresponds to a proximal signal of regulatory success; eudaimonia reflects the stabilization of identity-level priors that organize goals and commitments over time; and psychological richness can be understood as epistemic foraging, that is, the active expansion and refinement of the generative model through engagement with informative experience. From this perspective, both mindfulness and psychotherapy can be conceptualized as interventions on precision-weighting and model updating: mindfulness modulates precision by relaxing overly rigid expectations and increasing tolerance for uncertainty, whereas psychotherapy supports cross-level integration and the reorganization of maladaptive priors. Together, these processes promote epistemic resilience and adaptive regulation across contexts and timescales. At the same time, it is important to acknowledge that Bayesian active inference and the Free Energy Principle have been the subject of extensive debate, particularly concerning their falsifiability, biological plausibility, and whether they offer genuinely mechanistic explanations rather than high-level descriptive frameworks (e.g., Raja et al., 2021; Aguilera et al., 2021; Mann et al., 2022). In the present work, these approaches are not treated as comprehensive or empirically complete theories of brain function. Instead, they are adopted as a formal and integrative framework for characterizing regulatory processes across multiple levels of analysis. Crucially, the claims advanced here do not depend on the full empirical validation of the Free Energy Principle as a general theory of brain function. Rather, they draw on more circumscribed and operational components of the framework—such as hierarchical predictive processing, precision-weighting, and policy selection—which are increasingly supported within computational neuroscience and cognitive science (see, e.g., Kanai et al., 2015; Sharp et al., 2023; Hohwy, 2020).

From this perspective, the framework serves as a unifying formalism within a broader account of happiness, health, and psychological well-being, integrating otherwise fragmented theoretical perspectives and processes—spanning perception, action, affect, and learning—within a common inferential architecture, while remaining open to ongoing empirical testing and theoretical refinement.

## Data Availability

The original contributions presented in this study are included in this article/supplementary material, further inquiries can be directed to the corresponding author.
